# Maternal blood folate status during early pregnancy and occurrence of autism spectrum disorder in offspring: a study of 62 serum biomarkers

**DOI:** 10.1186/s13229-020-0315-z

**Published:** 2020-01-16

**Authors:** Olga Egorova, Robin Myte, Jörn Schneede, Bruno Hägglöf, Sven Bölte, Erik Domellöf, Barbro Ivars A’roch, Fredrik Elgh, Per Magne Ueland, Sven-Arne Silfverdal

**Affiliations:** 1 Department of Clinical Sciences, Pediatrics, Umeå University, Umeå, Sweden; 2 Department of Radiation Sciences, Oncology, Umeå University, Umeå, Sweden; 3 Department of Clinical Pharmacology, Pharmacology and Clinical Neurosciences, Umeå University, Umeå, Sweden; 4 Department of Child and Adolescent Psychiatry, Umea University, Umeå, Sweden; 5 Center of Neurodevelopmental Disorders (KIND), Centre for Psychiatry Research, Stockholm, Sweden; 6 Department of Women’s and Children’s Health, Karolinska Institutet & Child and Adolescent Psychiatry, Stockholm Health Care Services, Region Stockholm, Stockholm, Sweden; 7 Curtin Autism Research Group, School of Occupational Therapy, Social Work and Speech Pathology, Curtin University, Perth, WA Australia; 8 Department of Psychology, Umeå University, Umeå, Sweden; 9 Department of Clinical Microbiology, Umeå University, Umeå, Sweden; 10 Bevital AS, Department of Clinical Science, University of Bergen, Bergen, Norway; 11 Department of Medical Biochemistry and Pharmacology, Haukeland University Hospital, Bergen, Norway

**Keywords:** Autism, Pregnancy, One-carbon metabolism, Folate, Vitamin B, Vitamin D, Vitamin A, Inflammation

## Abstract

**Background:**

Autism spectrum disorder (ASD) evolves from an interplay between genetic and environmental factors during prenatal development. Since identifying maternal biomarkers associated with ASD risk in offspring during early pregnancy might result in new strategies for intervention, we investigated maternal metabolic biomarkers in relation to occurrence of ASD in offspring using both univariate logistic regression and multivariate network analysis.

**Methods:**

Serum samples from 100 women with an offspring diagnosed with ASD and 100 matched control women with typically developing offspring were collected at week 14 of pregnancy. Concentrations of 62 metabolic biomarkers were determined, including amino acids, vitamins (A, B, D, E, and K), and biomarkers related to folate (vitamin B_9_) metabolism, lifestyle factors, as well as C-reactive protein (CRP), the kynurenine-tryptophan ratio (KTR), and neopterin as markers of inflammation and immune activation.

**Results:**

We found weak evidence for a positive association between higher maternal serum concentrations of folate and increased occurrence of ASD (OR per 1 SD increase: 1.70, 95% CI 1.22–2.37, FDR adjusted *P* = 0.07). Multivariate network analysis confirmed expected internal biochemical relations between the biomarkers. Neither inflammation markers nor vitamin D_3_ levels, all hypothesized to be involved in ASD etiology, displayed associations with ASD occurrence in the offspring.

**Conclusions:**

Our findings suggest that high maternal serum folate status during early pregnancy may be associated with the occurrence of ASD in offspring. No inference about physiological mechanisms behind this observation can be made at the present time because blood folate levels may have complex relations with nutritional intake, the cellular folate status and status of other B-vitamins. Therefore, further investigations, which may clarify the potential role and mechanisms of maternal blood folate status in ASD risk and the interplay with other potential risk factors, in larger materials are warranted.

## Background

Autism spectrum disorder (ASD) is a developmental condition characterized by social communication and interaction challenges alongside with repetitive, stereotypic, restricted interests, and behaviors causing different levels of disability [1]. Over the last two decades, the prevalence of ASD has increased considerably and is now estimated to be 1–3% in high-income countries [2–4]. A plethora of factors are discussed as an explanation for this trend, among them changes in the diagnostic criteria, increased awareness and access to care, improved services, but also changes in environmental conditions, micronutrient status, and maternal lifestyle [5–7]. Genome sequencing data indicate hundreds of potential ASD risk genes, both rare and common variants [7–9]. Further, the systemic and central nervous system physiology of ASD suggests a possible role of oxidative stress, neuroinflammation, and mitochondrial alterations that are likely to be influenced by gene-environment interactions [10, 11]. In individuals with a genetic predisposition, a range of environmental factors have been proposed to increase ASD risk [7, 12]. Among them are variations in maternal metabolism at pregnancy [13, 14]. Beside maternal metabolism, adverse health conditions during early pregnancy such as viral infections, inflammation, and immune activation are considered as risk factors for psychiatric disorders in the offspring [15–19]. Environmental factors may act via epigenetic mechanisms that are potentially modifiable and preventable [20]; therefore, the understanding of such risk factors may indicate a path to reduce incidence of ASD onset.

Previous studies were usually limited to one or two metabolic factors such as body-mass index and result of maternal diet and fatty acid metabolism [21–23], smoking [24] vitamin D [25–34] and folate supplementation [35–43], paracetamol [44, 45], valproic acid, thalidomide, and antidepressants [13, 46, 47], and phthalates [48]. Maternal defects in one-carbon metabolism have also been proposed to influence ASD risk [49–52]. So far, however, no single factor has been identified to play a leading role in the development of ASD [53–55], giving support to the notion that autism has a complex and multifactorial origin [56].

In the present paper, we investigate a comprehensive panel of 62 serum biomarkers related to one-carbon metabolism, inflammation, and life-style and fat-soluble vitamins in first-trimester maternal blood samples. To the best of our knowledge, there are no previous studies that have simultaneously investigated a large spectrum of metabolic markers in maternal blood in relation to ASD risk in the offspring. Based on published literature, our primary hypothesis was that low maternal folate and vitamin D status and increased levels of markers of inflammation and immune activation such as C-reactive protein (CRP), neopterin, and a higher kynurenine-tryptophan ratio (KTR) might be associated with increased risk of ASD in the offspring.

The potential impact of vitamin D on ASD onset has been discussed extensively during the last two decades [12, 57, 58]. Connection between vitamin D and ASD onset give facts that individuals with ASD have been observed to suffer from vitamin D deficiencies [59–62], and vitamin D supplementation may improve their socio-psychological status [63, 64]. Highest fraction of children with ASD are born in the end of the summer, that menaces that mothers had relatively lower vitamin D levels at the first and second trimester of pregnancy [65]. Numerous studies associate low vitamin D status and/or lack vitamin D formation and supplementation during pregnancy with an elevated risk of ASD in offspring in Sweden [32–34] and world-wide [25–29, 31]. In animals, vitamin D insufficiency leads to aberrant brain development and autism-resembling behavior [66]. The ability of vitamin D to downregulate substances associated with a risk of ASD occurrence, such as free radicals and heavy metals [67] and neurologically harmful cytokines [68], to increase cellular levels of the anti-oxidant glutathione [69] and its association with the ability of the brain to recover after damage [70] connects vitamin D deficiency with a possibility of increased risk of ASD onset. ASD is supposed to be an inflammation-connected disorder [18], with elevated cytokine levels in the patients’ blood [71–73], altered mononuclear cell activation [74, 75], astrocyte activation, and neuroinflammation [76–80]. Vitamin D has an anti-inflammatory activity [81–83], downregulates inflammation both in immune and nervous systems [84], and normalizes macrophage activity in peripheral blood in persons with ASD [85]. Even direct influence of vitamin D on gene expression cannot be ruled out as receptors for vitamin D are broadly expressed throughout developing brain tissue [86] and are known to regulate the synthesis of many proteins involved in mammalian brain development [87, 88].

Folate is a collective term for folic acid derivatives. Pre-, periconceptional and first trimester pregnancy folic acid supplementation is successfully used to prevent neural tube defects [89]. Folate is transported into the cell by proton-coupled folate transporter, reduced folate carrier 1 and folate receptors (FRs). Four folate receptors have been discovered until now. The folate receptor subclass 1 (FR1), also named FRα, is expressed during development in placental, choroid, and neural plate cells. Mouse embryos homozygote for the knock-out FR*α* gene develop neural-tube defects and die before birth [90]. Folate is involved in transmission of one-carbon groups and thus synthesis of amino acids, nucleotides, neurotransmitters synthesis, nuclear acids, and proteins. Folate-dependent homocysteine remethylation connects folate with the transsulfuration pathway [91, 92]. Beside its metabolic function, folate also has a direct influence on immune system modulation [93, 94] and on gene expression regulation through FRα [95, 96]. In such way, the alteration of cell folate levels may trigger the onset of ASD through numerous processes in the developing brain from DNA synthesis and cell proliferation to neurotransmitters and other cell signal molecule synthesis, lipid synthesis, membrane formation, and myelination. Maternal folate levels during pregnancy may even cause onset of ASD in children through immune system modulation. More than a decade ago, it was suggested that folic acid use by the mothers could been one of the factors that increase the risk of ASD in children [97], an observation confirmed by some [42, 43, 96, 98] but rejected by other [35–39, 98] studies. It is important, therefore, to explore the question further.

The sensitive period for ASD onset in humans is yet not determined. As ASD is known to be accompanied by a modified brain structure and connectivity [99, 100], the risk for ASD is associated mostly with unfavorable events in the second and third trimester of pregnancy when the connections between neurons establish [100, 101]. However, there are suggestions that alterations in fetal brain development at the end of the first–beginning of the second trimester of pregnancy, a window of neuron production and migration inside the developing brain, may also cause ASD onset [13, 102] Therefore, we consider it possible to use the pre-diagnostic maternal blood samples collected during week 14 of pregnancy for our study.

## Methods and material

### Study population

**Fig. 1:**
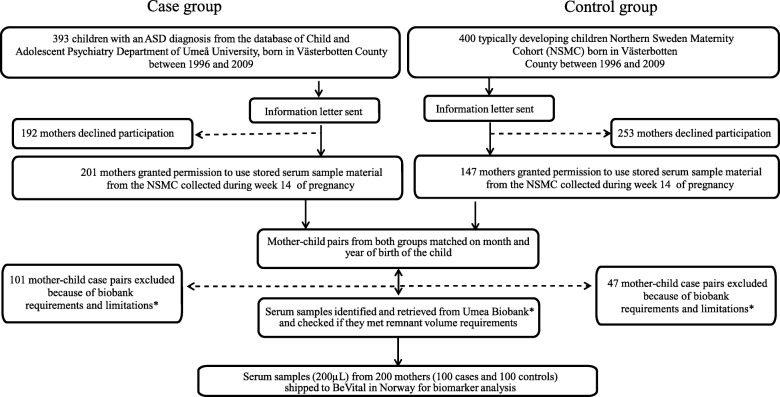
Study design

Children born in Västerbotten County, northern Sweden, with mothers who had been residing in Sweden during the whole pregnancy period were eligible for the study. A total of 393 children with an ICD-10 based ASD diagnosis, born between 1996 and 2009 (Fig. 1 and Table 1), were identified from medical records from the Child and Adolescent Psychiatric Clinic in Västerbotten County. The diagnostic procedure followed standards from Child Neuropsychiatric specialized teams mainly working in Child and Adolescent Psychiatry clinics and Child rehabilitation centers. The diagnostic process included medical history, current and past symptomatology, and functional abilities according to parent and teacher interviews as well as rating scales, child observation data, and psychological and medical tests. Children were diagnosed according to DSM-IV [1] and met the ICD-10 criteria for F 84.0 (Infantile autism), or F 84.5 (Asperger syndrome). Permission for using stored pre-diagnostic blood samples was granted from 201 women with an offspring diagnosed with ASD (Fig. 1). From the population register of Västerbotten County, 400 typically developing children, born between the years 1996 to 2009, were identified and asked to participate in the study, and 147 mothers granted permission to use blood samples (Fig. 1). Because the present investigation was novel and feasibility of the sample material for analysis of the proposed biomarkers had not been tested before, the biobanks steering committee gave access to serum samples from only 100 pregnant women who gave birth to a child diagnosed subsequently with ASD (74 cases of infantile autism, and 26 cases with Asperger syndrome) and from 100 pregnant mothers giving birth to a typically developing child. Further requirements were that the remnant volume in sample tubes should be at least 700 μL and that a maximum of 200 μL serum could be used for the present project. Groups were matched by year and month of childbirth.

**Table 1:** Baseline characteristics

	Cases (*n* = 100)		Controls (*n* = 100)		
	*n*	Median (IQR^a^) or %	n	Median (IQR) or %	*P*^b^
Mother’s age (years)	100	31 (28–34)	100	30 (26–33)	0.02
Year of blood serum sampling	100	2003 (2001–2005)	100	2002 (1999–2005)	0.07
Month of birth, child	100	7 (4–10)	100	8 (4–10)	0.50
Child sex, boy	76	76%	78	78%	0.87
ASD diagnosis					
Infantile autism (F84.0)	74	74%			
Asperger’s syndrome (F84.5)	26	26%			

^a^Interquartile range (IQR), 25–75th percentile

^b^Test for difference with Mann-Whitney *U* test (continuous variables) or Chi-square test (categorical variables)

### Collection, storage of blood serum samples

The serum samples used in this study were obtained from the Northern Sweden Maternity Cohort (NSMC) founded in 1975 [103]. The serum samples were routinely collected for testing of systemic infections and rubella IgG antibody titers at the first midwife visit at one of the more than 30 primary health care centers in Västerbotten County. The time point of the first visit is at the discretion of the patients and occurs in most cases between weeks 7 and 18 centering around week 14 [103, 104]. As we did not have access to the exact gestational age at blood sampling, we assigned all patients to week 14 [104]. Samples were exposed to room temperature and initial storage in refrigerators at 4 °C at the primary care centers for variable periods and periodically shipped, frozen, to a central repository at Umeå University Hospital, where they are analyzed and the leftovers were stored at − 20 °C [103, 104].

### Biochemical analyses

Serum samples were analyzed at Bevital AS (Bergen, Norway) [105]. We analyzed a total of 62 biomarkers with focus on one-carbon metabolism including B-vitamins; lipid-soluble vitamins A, D, E, and K; markers of immune activation and inflammation such as CRP, KTR, and neopterin; and lifestyle factors (such as cotinine, a marker of nicotine exposure, and trigonelline, a marker of coffee consumption) (Additional file 4: Table S1). The different biomarkers were analyzed using liquid and gas chromatography–mass spectrometry methods [106–108], immuno-MALDI-TOF mass spectrometry [109], and microbiological methods [110, 111]. Details on analytes stability, assay characteristics, analytical performance, and reference ranges can be found elsewhere [108, 111–116]. Most likely due to non-optimal initial storage conditions (see above), as well as the limited total sample volume not allowing for repeat analysis, 13 biomarkers (5-formyl-tetrahydrofolate, pyridoxal 5′-phosphate [vitamin B6], pyridoxine, thiamine, thiamine monophosphate [vitamin B_3_], 3-hydroxykynurenine, anthranilic acid, 3-hydroxyanthranilic acid, picolinic acid, α-ketoglutaric acid, phylloquinone [vitamin K1], menaquinone-4 [vitamin K_2_], 25-hydroxyergocalciferol [25OH-vitamin D_2_]) could only be quantified in a limited number of cases (< 10% of the total number of available serum samples) and were consequently excluded from further statistical analyses (Additional file 4: Table S1). Unmetabolized folic acid was detected in small amount of samples (17%) and these samples were excluded from further statistical analyses of biomarkers because we assumed that that unmetabolized folic acid was an expression of higher folate exposure most likely due to high doses of supplemental folic acid. As there is no mandatory folic acid food fortification in Sweden and the majority of pregnancies are unplanned folic acid intake during early pregnancy might rather be an indicator of co-morbidities and not of natural periconceptional folic acid intake. In multivariate analyses, we further excluded methionine and acetamidobenzoylglutamate (determination was not possible in 41 and 33% of the samples, respectively). Cotinine, a marker of nicotine exposure, exhibits high storage stability [117]. We used serum concentrations > 85 nmol/L, as cut-off for active smoking, a values below as an indicator of passive and non-smoking. We also calculated the kynurenine/tryptophan ratio (KTR) as a marker of immune activation [118]. By oxidation processes during storage 5-methyl-tetrahydropholate (mTHF) is partly converted into 4-alfa-hydroxy-5-methyl-tetrahydrofolate (hmTHF). Therefore, for the further statistical analysis, we used the parameter ‘total folate’ which is the sum of detected mTHF and hmTHF [111].

In total, 45 out of the tested 62 biomarkers could be identified in the present sample material and were included in the final statistical analyses (Additional file 4: Table S1, Fig. 4).

### Statistical analysis

Mann-Whitney *U* test or Chi-square tests were used to test for differences in baseline characteristics between cases and controls. Correlations between metabolites were calculated with Spearman’s correlation coefficient on pair-wise complete observations.

Associations between serum biomarkers and ASD occurrence were estimated as odds ratios (ORs) per 1 standard deviation (SD) increase in biomarker concentrations using multivariable adjusted logistic regression. Estimates were adjusted for mother’s age, sampling year, child gender, serum cotinine, and serum total folate (as a marker for overall folate metabolism status). Estimates for vitamin D, the only biomarker showing seasonal differences (Additional file 2: Figure S2), were further adjusted according to half-year of blood sampling (April-September or October-March). *P* values were corrected for multiple testing by controlling the false discovery rate (FDR) according to the Benjamini and Hochberg method [119]. To evaluate potential differences in association by ASD type, we also estimated type-specific ORs for infantile autism and Asperger’s syndrome using multinomial logistic regression. Heterogeneity in association between the types was examined with Wald’s test.

To account for complex interrelations between the biomarkers, we estimated undirected Bayesian networks using machine learning. A Bayesian network is a graphical representation of all relations among a set of variables, depicted as nodes and edges. *Nodes* represent the variables (here biomarkers) while *edges* represent conditional dependencies between variables. Bayesian network analysis allows simultaneous modeling of multiple dependencies between potential risk factors and disease. Bayesian network analysis can be considered as an appropriate means to study interactions and relative contributions between different above mentioned components of maternal metabolism during early pregnancy and ASD [120]. The network models were estimated on discrete data, with serum biomarkers dichotomized into low/high groups, below/above the median of biomarker concentration distributions of the controls, and further included mother’s age, sampling year, sampling month, and sex of child. Networks were estimated using the Hill-climbing algorithm in the bnlearn R-package in 1000 bootstrap samples drawn from the data set [121]. The final network was achieved by averaging over the bootstrap networks, where an independent association (i.e., an edge between two nodes) was included if it was present in a frequency above an estimated threshold [120]. Association strength between the variables in the network was measured by the frequencies of an independent association in networks present in the 1000 bootstrap samples (a measure between 0, not present in any bootstrap network, and 100%, present in all 1000 bootstrap networks).

All computations were conducted in R v. 3.4.2 (R Foundation for Statistical Computing, Vienna, Austria.). Network visualizations were made in Cytoscape v. 3.2.1. All tests were two-sided and *P* values < 0.05 were considered significant.

### Ethics

The study was approved by the Regional Ethics Board at Umeå University, Sweden, 2013-06-18, Dnr 2013-66-31M. Through an information letter, written informed consent was obtained, and mothers gave permission for using stored early pregnancy blood samples for the purpose of the present study, and for collecting registered information.

## Results

### Baseline characteristics

From the NSMC, we got access to a total of 100 blood samples from mothers with occurrence of ASD in off-spring (case group) and 100 blood samples from the matched control group (Fig. 1). Baseline characteristics for cases and controls are presented in Table 1. The mothers of children later diagnosed with ASD were marginally older at the time of blood sampling compared to the mothers constituting the control group (31 vs. 30 years).

Unmetabolized folic acid was detected in a larger, however statistically insignificant, number of mothers in the case group compared to controls (22% and 12% respectively, *P* = 0.09) (Additional file 4: Table S1).

Serum vitamin D levels showed seasonal variations and were slightly higher in blood samples collected during the months from July to October (Additional file 2: Figure S2). No other biomarkers exhibited clear seasonal differences. For unknown reasons, several biomarkers differed markedly by sampling year, in particular before and after 2001 (Additional file 2: Figure S2). However, seasonal and year associations were preserved and essentially the same in cases and controls (Additional file 1: Figure S1 and Additional file 2: Figure S2).

Levels of kynurenine, kynurenic acid, and quinolinic acid were slightly higher in male pregnancies (approximately 10% higher concentrations compared to mothers pregnant with girls, *P* = 0.03, 0.04, and 0.06, respectively). No other significant differences by sex of the offspring were observed.

**Fig. 2:**
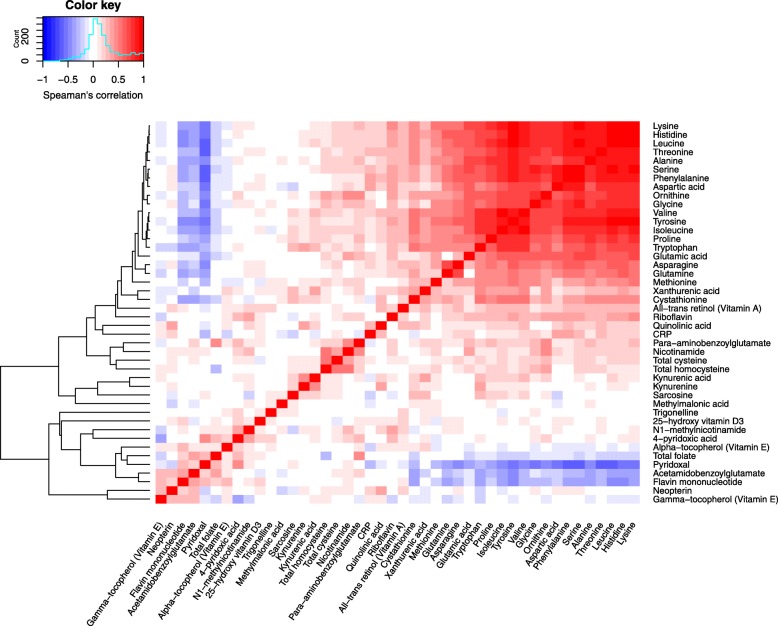
Spearman’s correlations between analyzed serum biomarkers with a hierarchical cluster analysis based on the correlations

Spearman’s correlations with a hierarchical cluster analysis between the serum biomarkers are presented in Fig. 2. Amino acids, such as lysine, serine, or glycine, showed strong, positive correlations. These amino acids were negatively correlated with B-vitamins (e.g., total folate and pyridoxal), and weakly positively associated with CRP. Vitamin D did not show strong associations with any of the markers (Fig. 2).

### Associations between biomarkers and ASD

**Fig. 3:**
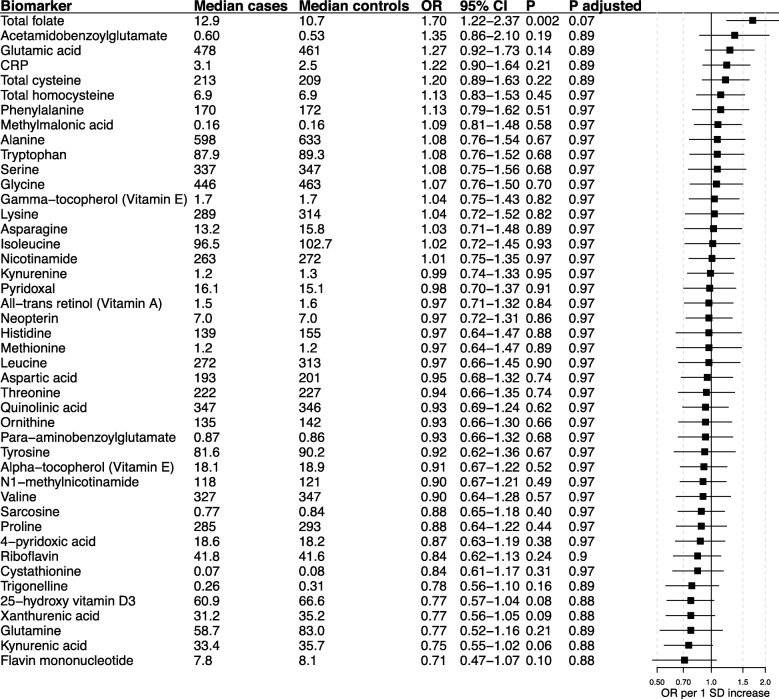
Odds ratios (ORs) for ASD risk by 1 standard deviation (SD) increase in biomarkers levels. ORs were calculated using logistic regression adjusted for mother’s age at sampling, year of sampling, sex of child, and serum cotinine and mTHF levels. Vitamin D_3_ was further adjusted for month of sampling (light/dark months). Median serum concentration levels in cases and controls are presented in nmol/L for biomarker kynurenic acid; 25-hydroxy vitamin D_3_; xanthurenic acid; trigonelline; cystathionine; 4-pyridoxic acid; quinolinic acid; pyridoxal; neopterin; nicotinic acid; para-amino benzoylglutamate; acetamidobenzoglutamate; hmTHF; mTHF;and in mg/l for CRP and all remaining biomarkers

Adjusted ORs for ASD occurrence per 1 SD increase in biomarker levels are presented in Fig. 3. Higher early pregnancy total folate levels were associated with an increased probability of having a child with ASD (OR per 1 SD increase: 1.70, 95% CI 1.22–2.37, *P* = 0.002). Taking multiple testing into account, weak evidence remained (FDR adjusted *P* = 0.07). No other biomarker showed any significant association with occurrence of ASD, including CRP (OR per 1 SD increase: 1.20, 95% CI 0.89–1.61, *P* = 0.24), the kynurenine/tryptophan ratio (OR 0.91, 95% CI 0.66–1.26, *P* = 0.58), neopterin (OR per 1 SD 0.97, 95% CI 0.72–1.31, *P* = 0.86), and vitamin D levels (OR per 1 SD increase: 0.78, 95% CI 0.58–1.08, *P* = 0.11). Active smoking, defined as serum cotinine > 85 nmol/l, was not associated with ASD risk in the present material (OR 1.21, 95% CI 0.51–2.88, *P* = 0.66). There was no significant difference in overall biomarker profile between cases and controls (Additional file 3: Figure S3). Risk estimates for the total folate-ASD association were similar for infantile autism and Asperger’s syndrome (ORs 1.69 and 1.68, P-heterogeneity = 0.99, Additional file 5: Table S2). No other biomarker displayed any clear difference in ASD association by type.

### Multivariate Bayesian network analysis

**Fig. 4:**
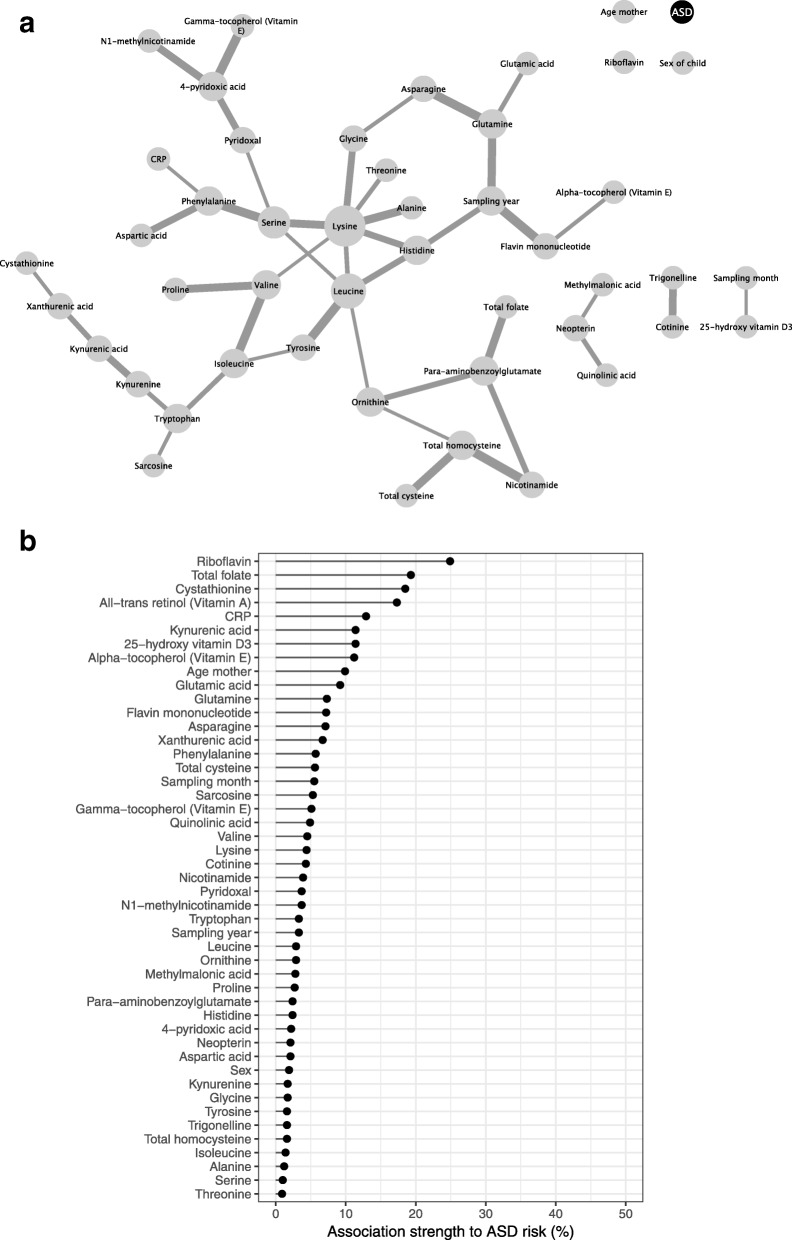
**a** Bayesian network of serum biomarkers and background information variables estimated by a Hill-climbing algorithm and averaged over 1000 bootstrap samples. A line between two variables indicates an association independent of all other variables in the network. Line thickness corresponds to association strength measured as proportion of times an association was present in 1000 bootstrap sample networks (a thicker line indicates a stronger association). Node size corresponds to the number of connections. The network was estimated using discrete data (with biomarkers divided into low/high groups, with cut-off defined by the median biomarker concentrations in the controls). **b** Association strengths to ASD risk for each biomarker measured as proportion of times an association was present in 1000 bootstrap sample networks

The Bayesian network depicting independent associations between all biomarkers and background variables is presented in Fig. 4a. The associations among the biomarkers illustrated in the network were largely in line with expected biochemical relations. For instance, the interconnectedness between circulating amino acids, associations between circulating metabolites in the kynurenine pathway (tryptophan, kynurenine, kynurenic acid, and xanthurenic acid), and the associations between coffee and smoking biomarkers trigonelline and cotinine. Independent associations between occurrence of ASD and all variables except for riboflavin and total folate were, however, generally weak (Fig. 4b), and did not reach the threshold for inclusion of 50%.

## Discussion

This case-control study with pre-diagnostic, first trimester maternal blood samples is the first of its kind to investigate a comprehensive panel of biomarkers reflecting vitamin status (including folate, and vitamin D), lifestyle (smoking and coffee consumption), and inflammation and immune activation (CRP, kynurenine, kynurenic acid, and quinolinic acid, KTR and neopterin) in relation to the occurrence of ASD in the offspring. The other characteristics of the present study is that biomarkers were analyzed in individual, pre-diagnostic maternal blood samples and not estimated based on register data or food frequency questionnaires [21–32, 35–46]. The majority of blood samples were taken at week 14, but ranged between week 7 and week 18 in the total cohort. Among cases, there was no indication of differences in the biomarker relationships between the ASD subtypes, infantile autism, and Asperger syndrome, indicating that results are probably valid across the autism spectrum and independent of IQ. In the entire sample, there were sex-dependent differences in tryptophan metabolism: kynurenine, kynurenic acid, and quinolinic acid were higher in serum of mothers pregnant with boys (approximately 10% higher than in mothers pregnant with girls). Total maternal folate serum levels at pregnancy were positively associated with the occurrence of ASD in children, although the association lost significance when adjusted for other factors. There were no associations between maternal vitamin D, life-style , and markers of inflammation or immune activation with ASD occurrence in children. Despite non-optimal initial storage conditions of samples and failure to determine the concentrations of some unstable metabolites, mostly from the vitamin B group, multivariate Bayesian network analysis (Fig. 4a) showed an expected relationship between biomarkers that conform the validity of analyses [105]. The validity of the analytical results in the present study is strengthened by the fact that the majority of biomarkers were found within the expected concentration ranges and that the anticipated subtle seasonal variations for vitamin D, folate, and CRP [105, 122] could be proved in our material.

Increased serum levels of kynurenine, kynurenic acid, and quinolinic acid were seen in mothers pregnant with boys both with and without ASD. These substances belong to the kynurenine pathway of tryptophan metabolism forming kynurenine that is further metabolized to kynurenic or quinolinic acid [123]. The sex difference cannot be explained simply by coincidental increased tryptophan consumption in male pregnancies, as male pregnancies are also characterized by slightly elevated kynurenine/tryptophan, kynurenine/kynurenic acid, and kynurenine/quinolinic acid ratios (data not shown). The human placenta as well as the uterus express indolamine-2,3-dioxygenase 1 (IDO1), an enzyme that convert tryptophan to kynurenine, believed attenuate anti-fetal immune response. IDO1 activity increases gradually from the first to third trimester of pregnancy with an increase in kynurenine levels in placenta and uterus approximately eight-fold toward the end of the pregnancy [124]. Therefore, increased kynurenine levels could be an expression of higher activity of IDO1 in male pregnancies and/or by a coincidental difference in fetal gestational age.

While kynurenine and kynurenic acid is believed to have an anti-inflammatory activity, quinolinic acid has proinflammatory and cyto- and neurotoxic properties [124, 125]. The presence of other neurotoxic substances in pregnancy [13, 18, 67], as well as immune system activation [15, 18, 19, 44, 45] are associated with a higher risk of ASD onset in offspring. Genetically predisposed male fetuses, being exposed for higher levels of neurotoxic substances such as quinolinic acid, may therefore run a higher risk of aberrant brain development compared to female fetuses. This difference between male and female pregnancies may partly explain the well-known three-fold difference in prevalence of ASD diagnosis between men and women [126].

Maternal vitamin D status showed no associations with ASD onset in children contrary to recently published Australian, Chinese, and Swedish studies [29, 31, 32]. However, in the latter Swedish study, maternal vitamin D levels were not measured specifically during pregnancy but were collected from patient healthcare files [32]. The protocols of the Chinese and Australian studies [29, 31] were more comparable with our protocol with the exception that the majority of blood samples were from gestational week 14 in our study, and at gestational week 12 and 18 in the Chinese and Australian studies, respectively. Notably, in the large Australian cohort, there were no overall associations between child diagnosis and maternal vitamin D levels. An association could only be seen when special features of psychical development where considered [31]. We did not have the information about participants’ social status, history of autoimmune disease, and psychiatric conditions in their families. All these factors may have strong impact on the likelihood of ASD occurrence in offspring [30–32] and failure to adjust for these parameters could have attenuated the expected associations between markers of inflammation and/or vitamin D in mothers blood and occurrence of ASD in children.

Two previous studies regarding connections between maternal CRP at mid-pregnancy (weeks 15–19) and risk of ASD in children [127, 128] showed opposite results. A statistically significant correlation between higher maternal CRP level and risk of ASD in children was found in a Finnish cohort [127], but in a comparable population from the USA, the correlation between maternal CRP and ASD in children was inversed [128]. These contradictory results were theorized to be caused by the genetic differences of the studied populations [128]. The results of our study showed no relation between maternal CRP and the occurrence of ASD in children. This may be related to a five to six times smaller amount of participants included in our work. In the American study, for example, there was also no correlation between maternal CRP and risk of ASD in children in the smallest of two subgroups [128].

The present blood specimens originated from the NSMK, a material originally collected for routine rubella serology testing. In the total cohort, gestational age ranged from 7 to 18 weeks. With respect to variations in menstrual cycle, duration of the follicular phase, and timing of conception, the fetal age may additionally vary by 3 weeks at the time of blood sampling [129]. Unfortunately, we do not have information about the exact time of collection of blood samples in our cohort, but variations in gestational age may have caused increased variability in our material and reduced the chances to find statistically significant associations between maternal CRP and/or vitamin D and occurrence of ASD in children.

Unlike other metabolites, high maternal folate status was positively associated with ASD occurrence in children. To the best of our knowledge, associations between maternal serum folate levels and ASD onset at this stage of pregnancy have not been reported before. Several previous studies have demonstrated an association of pre-, peri-, and postconceptional dietary folate intake with a decreased risk of ASD occurrence in offspring [35–39]. Folic acid supplementation in the first half of pregnancy was associated with decreased risk of ASD in children in USA [35], Norway [36], Canada [37], and Israel [38]. Contrary to the above mentioned, recent population-based studies in Denmark and Canada did not find any relationship between ASD risk and the use of folic acid supplementation by mothers between week − 4 to + 8 of pregnancy [40, 41].

It seems that maternal folate intake and risk of ASD in children may have more complicated relations. A Spanish study showed associations between decreased child psychomotor development and intake of more than 400 μg folic acid per day throughout pregnancy [42] and, very recently, in the Boston birth cohort, high folate and vitamin B_12_ vitamin blood levels at birth were associated with an elevated risk of ASD in children [43]. The present study is based on a population not exposed to nutritional folic acid. In Sweden, food is generally not fortified with folic acid (in contrast to the USA, where blood folate levels are much higher due to food fortification). Dietary folate intake and prevalence of folic acid supplementation during pregnancy are known to be generally low in Sweden [130]. The vitamin concentrations observed in our material are below the levels that were reported in the Spanish and American studies. Therefore, different mechanisms may lay behind the association between folate status and ASD onset in the present study as compared to the abovementioned Spanish and Boston cohorts.

The information about vitamin supplementation in most of the abovementioned studies was obtained from food frequency questionnaires, pharmacy registers, or interviews. The present study does not use data about vitamin supplementation, but associates folate concentration in maternal serum with the occurrence of ASD in children. Serum folate levels may not be directly related to the amount of dietary folate intake. Previously elevated maternal whole blood folate levels during the first half of pregnancy were associated with a decreased risk of ASD in children of women with epilepsy [39], but an association between low whole blood folate levels and ASD risk for offspring of healthy mothers during the same period of pregnancy was not found [37]. In the same study, maternal folate supplementation during pregnancy was associated with a decreased ASD risk in children [37]. Therefore, the increased blood/serum folate levels may not be strictly related to dietary folate intake, but be a result of metabolic specificity.

The association of ASD risk with elevated circulating folate observed in our study may reflect altered activities of enzymes involved on folate-dependent one-carbon metabolism and/or impaired cobalamin function [131]. Thus, increased total folate in serum may be an epiphenomenon rather than the cause of ASD onset. This contention is supported by the results from a recent study reporting no association between maternal folic acid supplementation and occurrence of ASD in children [40, 41]. Another possible explanation could be disturbed folate uptake from blood into cells. Recently, ASD onset has been related to FRα-autoantibodies, that seem to be prevalent in children with ASD and their relatives including their mothers [132–136]. Folate receptors alpha (FR훼)-autoantibodies prevent normal folate transport across the blood–brain barrier while normal content is observed in the blood [137] and may be a reason of folate insufficiency in cells with simultaneous high folate levels in blood.

In our study, unmetabolized folic acid was detected in a larger number of mothers in the case group compared to controls, suggesting that supplementation with folic acid around gestational week 14 was more common in mothers whose children later developed ASD. Folic acid taken as a supplement is rapidly converted to 5,10-methylenetetrahydrofolate (5,10-methlyTHF), and unmetabolized folic acid is not supposed to be found in serum in subjects taking less than 400 μg/day [138]. Due to the limited sample size, the association did not reach statistical significance, but one may speculate if these women may have been using folic acid supplementation due to events in their medical history, such as unsuccessful attempts to become pregnant, earlier pregnancies with neural tube defects, or similar conditions that may have triggered folic acid supplementation. Considering recent findings confirming the notion of the complex role of folic acid in neurodevelopment [97], such as the direct correlation between extremely high maternal blood folate/folic acid supplementation and risk of ASD in children [42, 43], the correlation between extremely high blood levels of unmetabolized folic acid at birth, and elevated risk of developing food allergy in later life [94], the ability of folate to differentially alter the expression of genes associated with ASD onset [96], the relations between folic acid supplementation during pregnancy, and health of the offspring deserve further investigation.

Currently, we are not able to draw firm conclusions about potential pathomechanisms that could explain the associations between the higher folate levels in mothers and ASD onset, but the finding certainly deserves further exploration.

In the present study, in contrast to earlier research [35, 36, 38, 40–42], folate status around week 14 of pregnancy was not estimated from register data or food frequency questionnaires but measured in pre-diagnostic maternal blood samples. The samples were collected during the first routine midwife visit, which coincides with a period supposed to be critical for autism onset [13, 102]. Ideally, blood sampling should have occurred during earlier pregnancy phases, which better corresponds with recommendations for folic acid supplementation, that is the periconceptional period and the first trimester of pregnancy [38]. On the other hand, studying the associations between folic acid supplementation and ASD occurrence was not the primary aim of the present investigation. Furthermore, the blood folate level differences might not solely be explained by the amount of orally taken folic acid but rather by the particular qualities of the maternal folate metabolism [137, 139].

The feasibility of the stored material for biomarker analysis was unknown at the time we planned the study. As a consequence, the present investigation was designed as a pilot study to test the viability of the biobank material for determination of metabolic markers and we were only granted limited access to the serum biobank material (a maximum 100 cases and 100 controls). Moreover, the sample volume was restricted to 200 μL by the biobank steering committee, and only samples with a remnant volume more than 700 μL were eligible for our study. These restrictions explain the limited sample size and made duplicate analyses impossible. As a result, the present investigation has limited statistical power and does not allow for detection of small effect size associations. Furthermore, for the reasons given above, we did not have access to blood samples from *all* mothers who consented to participate in the study (Fig. 1). However, the proportion of missing blood samples was equal for both cases and controls. Therefore, a selection bias is unlikely. The only matching criterion between cases and controls was the child’s year of birth, which further reduces the potential risk of bias and, in addition, the risk of group disparities due to storage artifacts. Indeed, pre-analytical variation due to non-optimal sample treatment and storage conditions are expected to attenuate rather than over-estimate of the observed relationships. Some of the biomarkers, such as compounds of B_6_ vitamin group (for example PLP), are known to be unstable during long-term storage [113], and could not be detected in our material, while others were excluded from further statistical analysis due to results grossly deviating from expected concentration ranges based on reference material from other populations [104] (Additional file 4: Table S1). Still, the majority of biomarkers were within the expected concentration ranges and followed the expected seasonal variations [105, 122], which strengthen the validity of our analytical results. Finally, we cannot completely rule out differences in socio-demographic factors, nutritional status, and lifestyle habits between cases and controls, which may have confounded our results.

## Conclusion

In the present study, we could not confirm postulated associations between ASD-risk in the offspring and maternal status of vitamin D, or markers of inflammation, but observed an unexpected difference in components of tryptophan metabolism in male and female pregnancies that may be linked to the 3:1 male-to-female prevalence typically found in ASD cohorts [126]. Opposed to our primary hypothesis that lower folate status may be associated with increased risk of ASD, we found weak evidence for an association between higher maternal folate status and occurrence of ASD in the offspring. Additional studies in larger cohorts utilizing optimally stored blood samples are warranted to elucidate the potential role of maternal folate status and folic acid supplementation during pregnancy in relation to ASD risk in the offspring.

## Supplementary information

**Additional file 1: Figure S1.** Seasonal variations of studied biomarkers. The smooth line was fitted with locally weighted scatterplot smoothing (loess). a All participants. b Case group. c Control group.

**Additional file 2: Figure S2.** Associations between sampling year and studied biomarkers. The smooth line was fitted with loess. a All participants. b Case group. c Control group.

**Additional file 3: Figure S3.** Biomarker levels in each study participant, with a hierarchical cluster analysis based on Euclidian distances with complete linkage. Each column represents a participant and each row a biomarker. Heat map colors represent standardized log-transformed biomarker levels in all participants (red) and below mean biamarker levels (blue).

**Additional file 4: Table S1.** List of serum metabolites that were analyzed and used in further analysis. ^1^Only sum of THM and mTHM were included in the analysis as ‘total folate’. ^2^The sums of the Asparagine and Aspartic acid and the f Glutamine and Glutamic acid levels were used in statistical analysis because of conversion between this forms in the stored samples. In the whole 45 biomarkers could be identified and included in the analysis.

**Additional file 5: Table S2.** Differences of biomarkers levels between mothers of children with infantile autism and Asperger’s syndrome.

## Data Availability

The datasets generated and analyzed during the current study are not publicly available due to private information they contain but are available from the corresponding author on reasonable request.

## Abbreviations

ASD: Autism spectrum disorder
CI: Confidence interval
CRP: C-reactive protein
DSM4: Diagnostic and statistical manual of mental disorders, 4-th edition
FDR: False discovery rate
FR1: Folate receptors subclass 1
FRs: Folate receptors
FRα: Folate receptors alpha
hmTHF: 4-Alfa-hydroxy-5-methyl-tetrahydrofolate
ICD: International statistical classification
KTR: Kynurenine/tryptophan ratio
mTHF: 5-Methyl-tetrahydropholate
MTHFR: Methylenetetrahydrofolate reductase
MTRR: Methionine synthase reductase
NSMC: Northern Sweden Maternity Cohort
OR: Odds ratio
SD: Standard deviation
tHcy: Total homocysteine
