# Supplementary figures and images for: Maternal blood folate status during early pregnancy and occurrence of autism spectrum disorder in offspring: a study of 62 serum biomarkers
Source: Mol Autism. 2020 Jan 16;11:7. doi: 10.1186/s13229-020-0315-z (PMC6964211; doi:10.1186/s13229-020-0315-z)

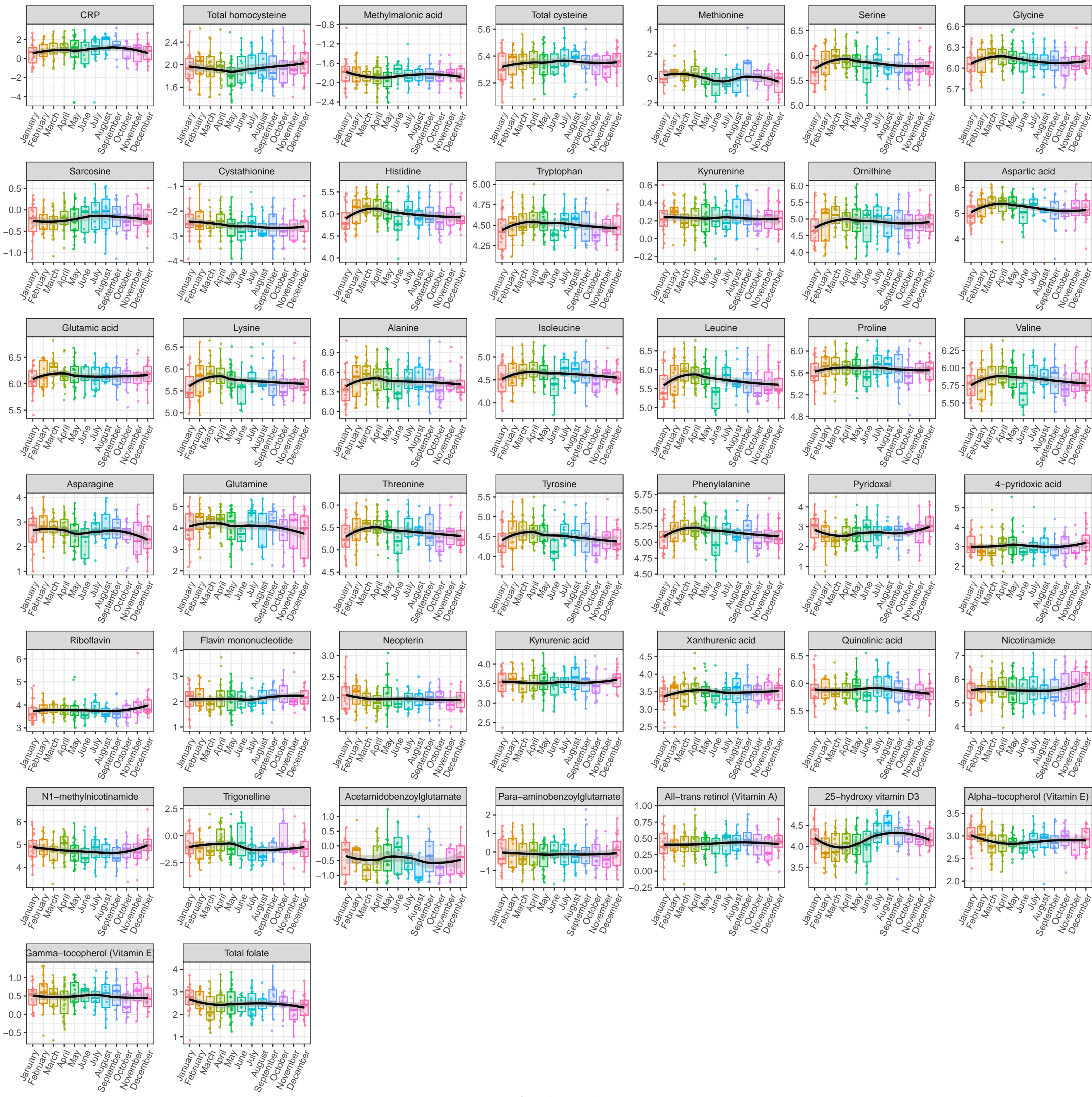

Sampling month

Supplement: Supplementary file 1 — Additional file 1: Figure S1. Seasonal variations of studied biomarkers. The smooth line was fitted with locally weighted scatterplot smoothing (loess). a All participants. b Case group. c Control group. [file 13229_2020_315_MOESM1_ESM.zip › supfig1a_all participants.pdf]

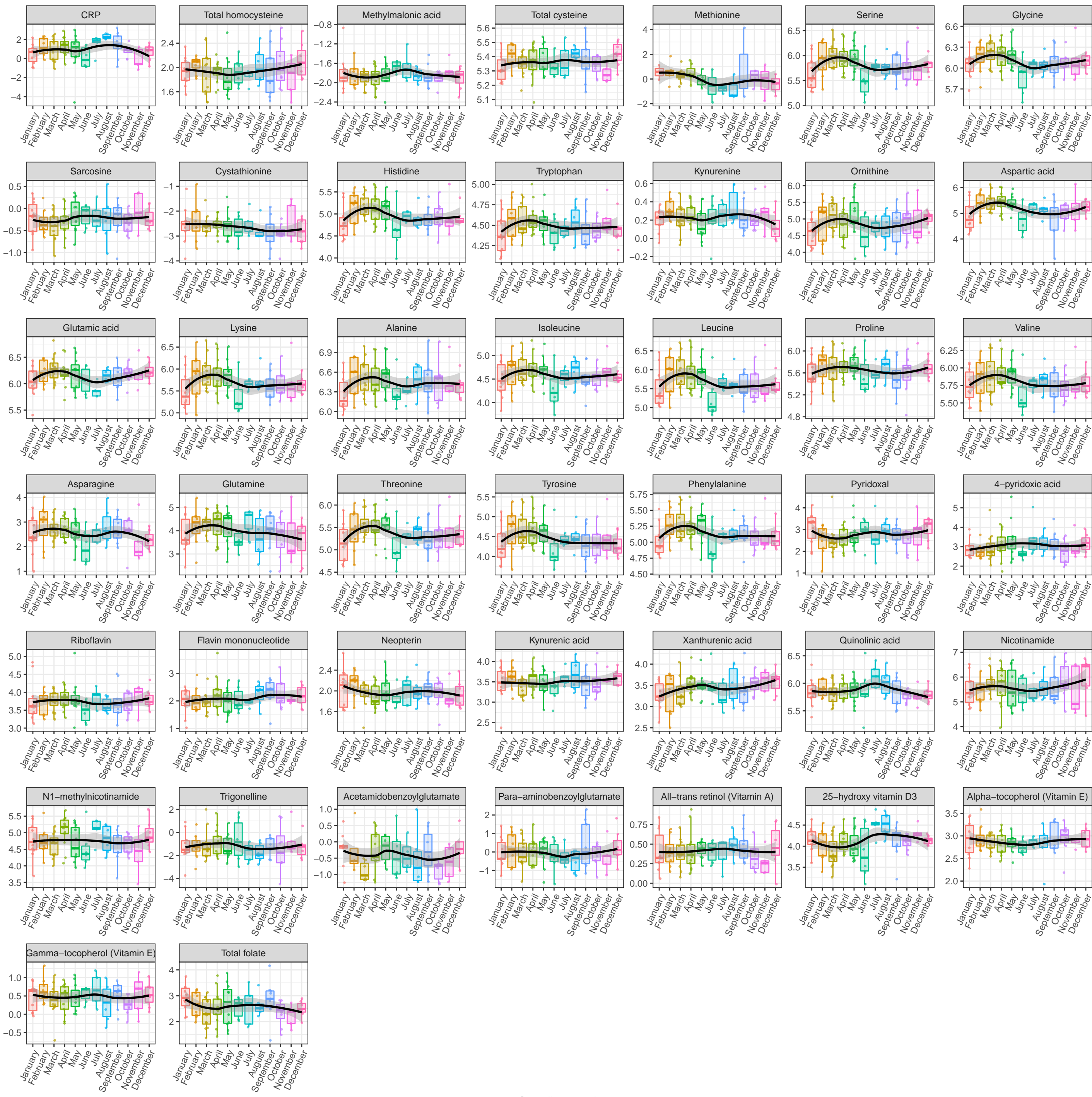

Supplement: Supplementary file 1 — Additional file 1: Figure S1. Seasonal variations of studied biomarkers. The smooth line was fitted with locally weighted scatterplot smoothing (loess). a All participants. b Case group. c Control group. [file 13229_2020_315_MOESM1_ESM.zip › supfig1b_ case group.pdf]

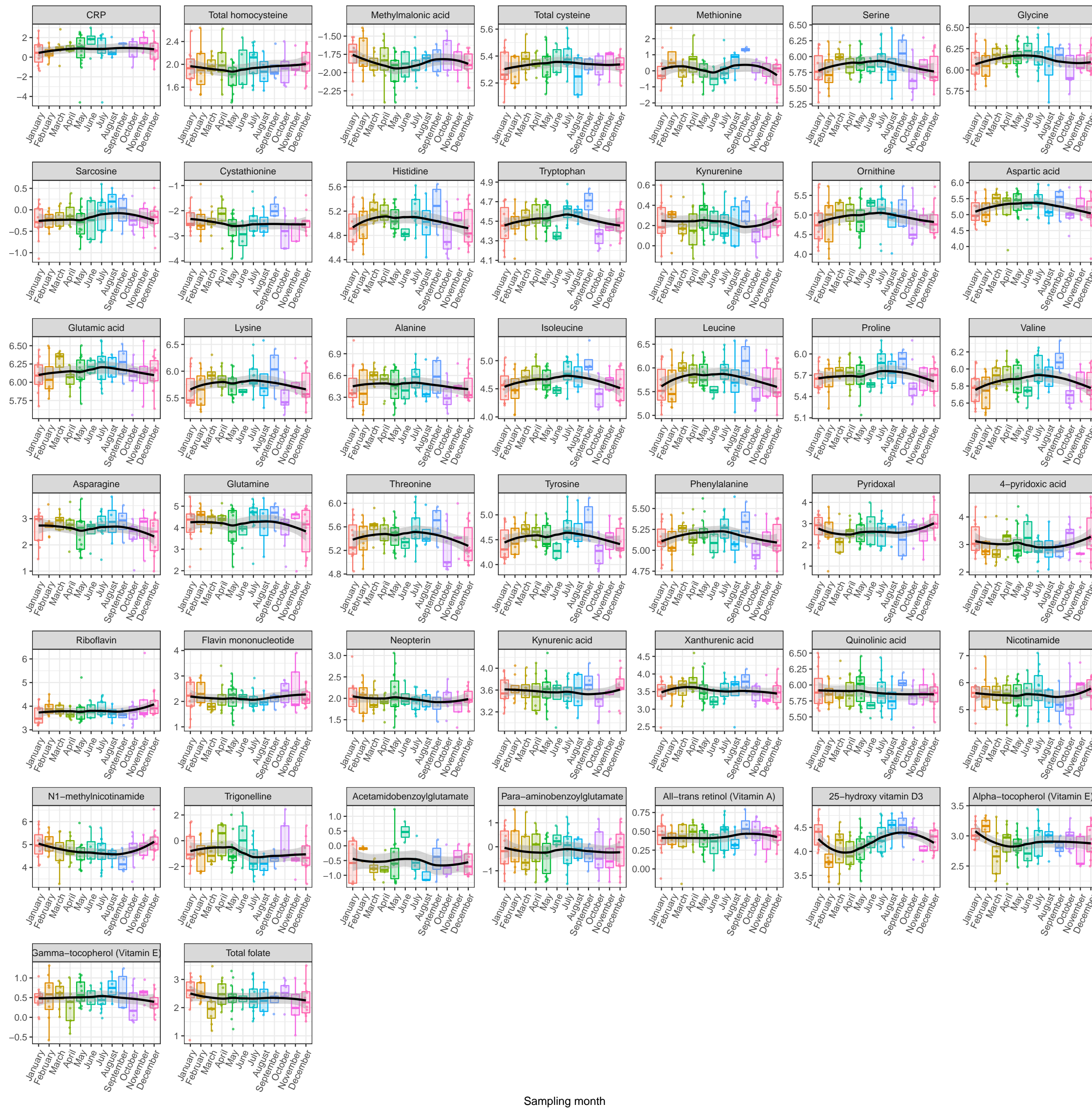

Supplement: Supplementary file 1 — Additional file 1: Figure S1. Seasonal variations of studied biomarkers. The smooth line was fitted with locally weighted scatterplot smoothing (loess). a All participants. b Case group. c Control group. [file 13229_2020_315_MOESM1_ESM.zip › supfig1c_controll group.pdf]

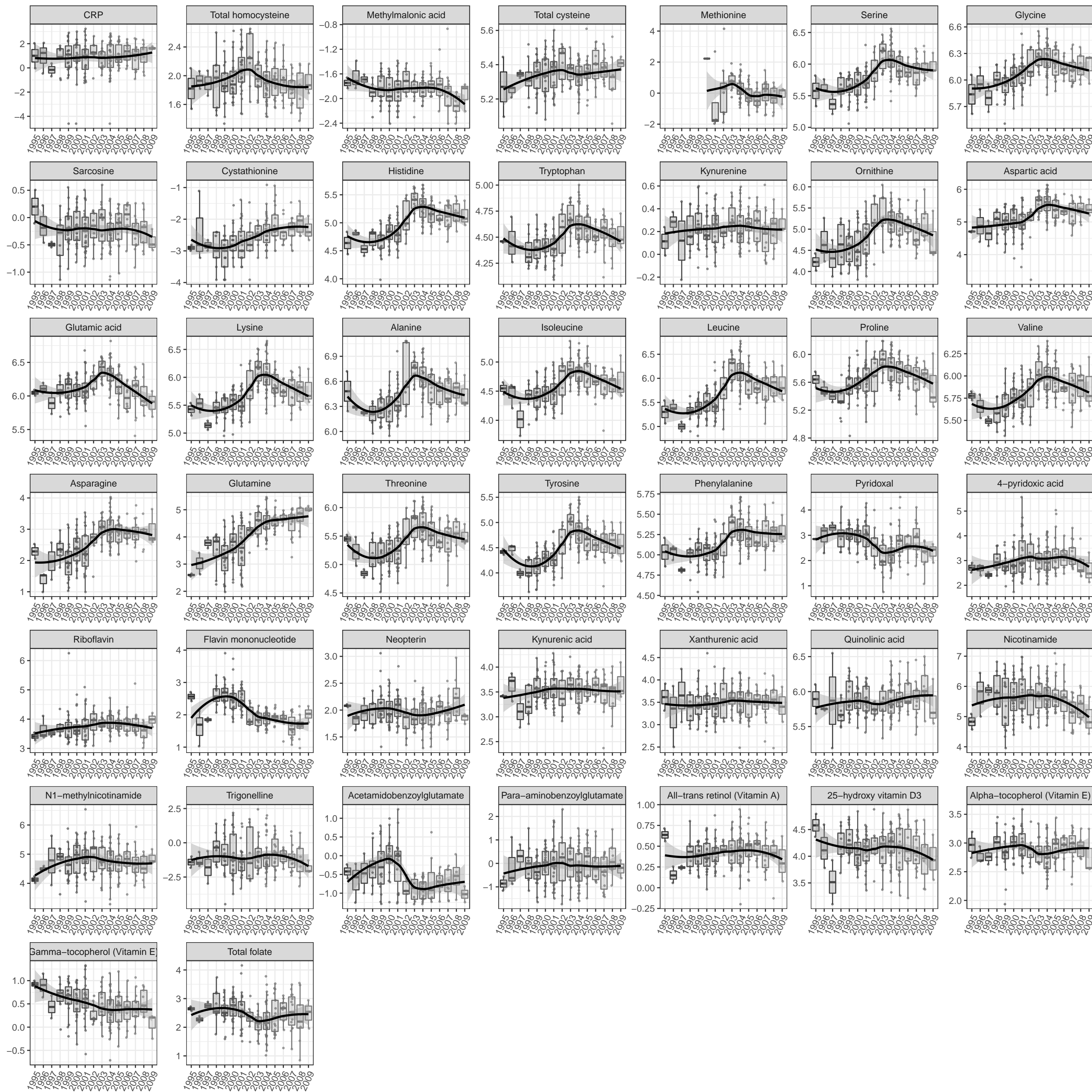

Sampling year

Supplement: Supplementary file 2 — Additional file 2: Figure S2. Associations between sampling year and studied biomarkers. The smooth line was fitted with loess. a All participants. b Case group. c Control group. [file 13229_2020_315_MOESM2_ESM.zip › supfig2a_all participants.pdf]

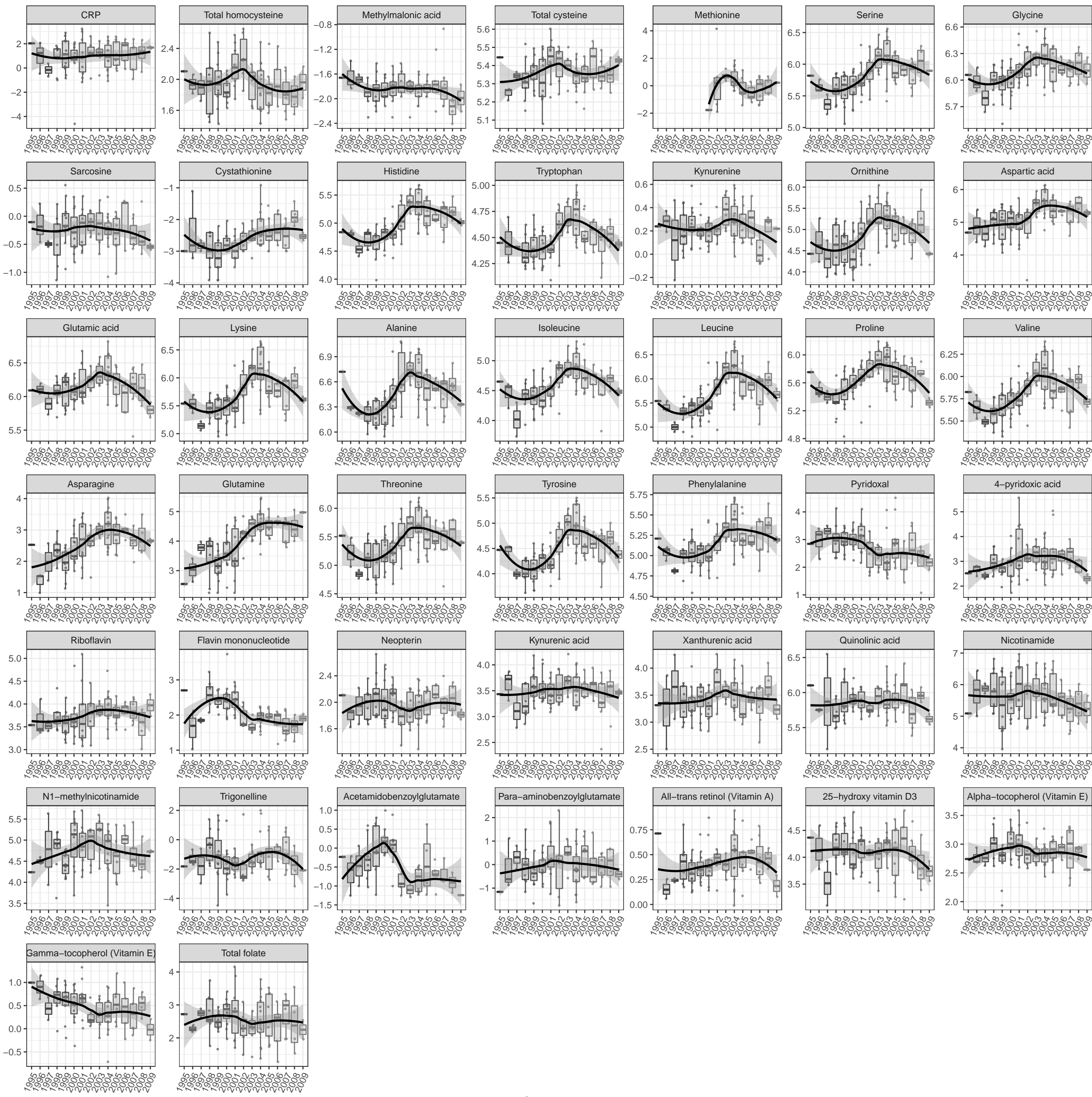

Sampling year

Supplement: Supplementary file 2 — Additional file 2: Figure S2. Associations between sampling year and studied biomarkers. The smooth line was fitted with loess. a All participants. b Case group. c Control group. [file 13229_2020_315_MOESM2_ESM.zip › supfig2b_cases group.pdf]

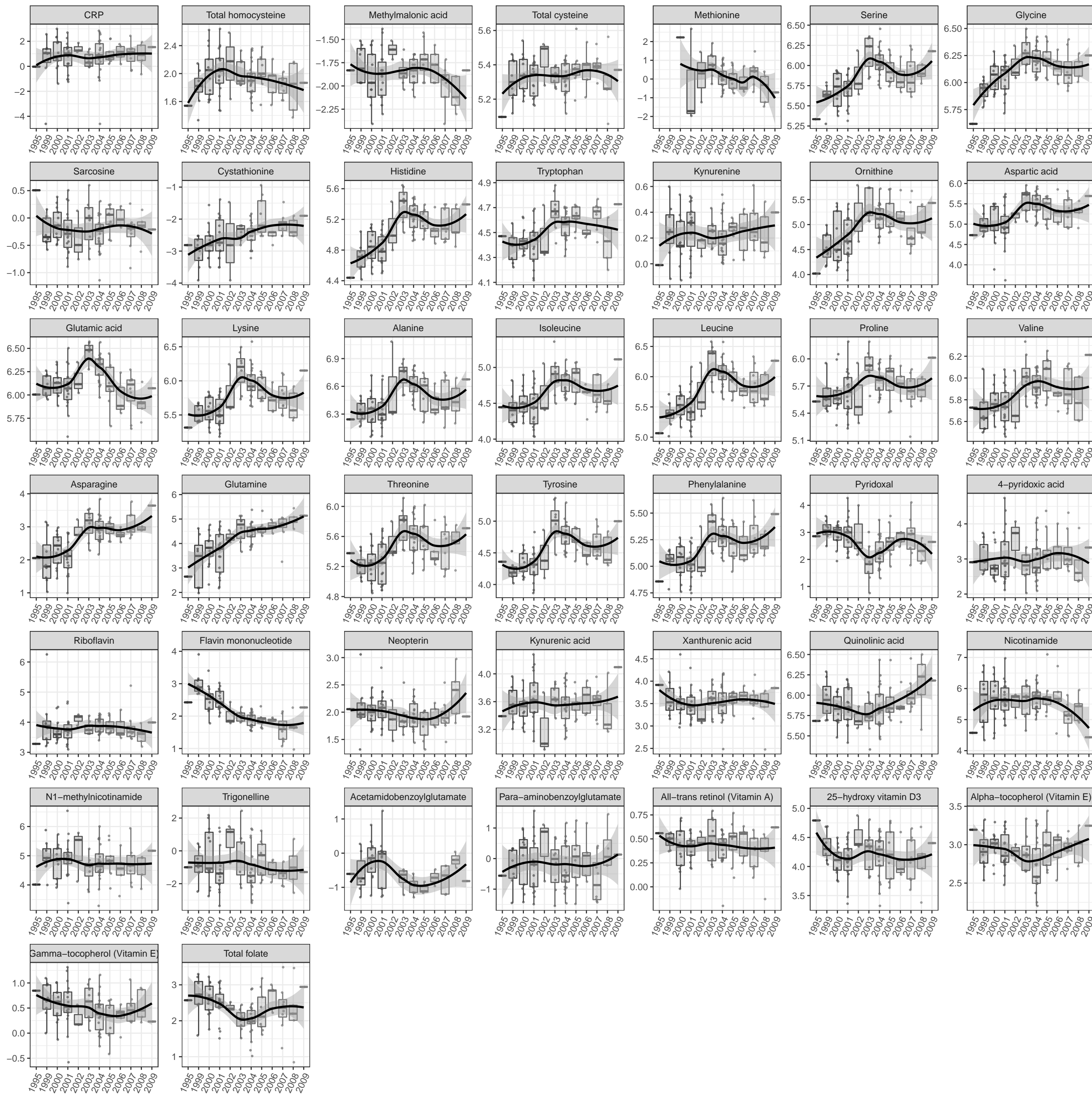

Sampling year

Supplement: Supplementary file 2 — Additional file 2: Figure S2. Associations between sampling year and studied biomarkers. The smooth line was fitted with loess. a All participants. b Case group. c Control group. [file 13229_2020_315_MOESM2_ESM.zip › supfig2c_control group.pdf]
