# Supplementary material for: Maternal blood folate status during early pregnancy and occurrence of autism spectrum disorder in offspring: a study of 62 serum biomarkers
Source: Mol Autism. 2020 Jan 16;11:7. doi: 10.1186/s13229-020-0315-z (PMC6964211; doi:10.1186/s13229-020-0315-z)
Supplement: Supplementary file 3 — Additional file 3: Figure S3. Biomarker levels in each study participant, with a hierarchical cluster analysis based on Euclidian distances with complete linkage. Each column represents a participant and each row a biomarker. Heat map colors represent standardized log-transformed biomarker levels in all participants (red) and below mean biamarker levels (blue). [file 13229_2020_315_MOESM3_ESM.pdf]

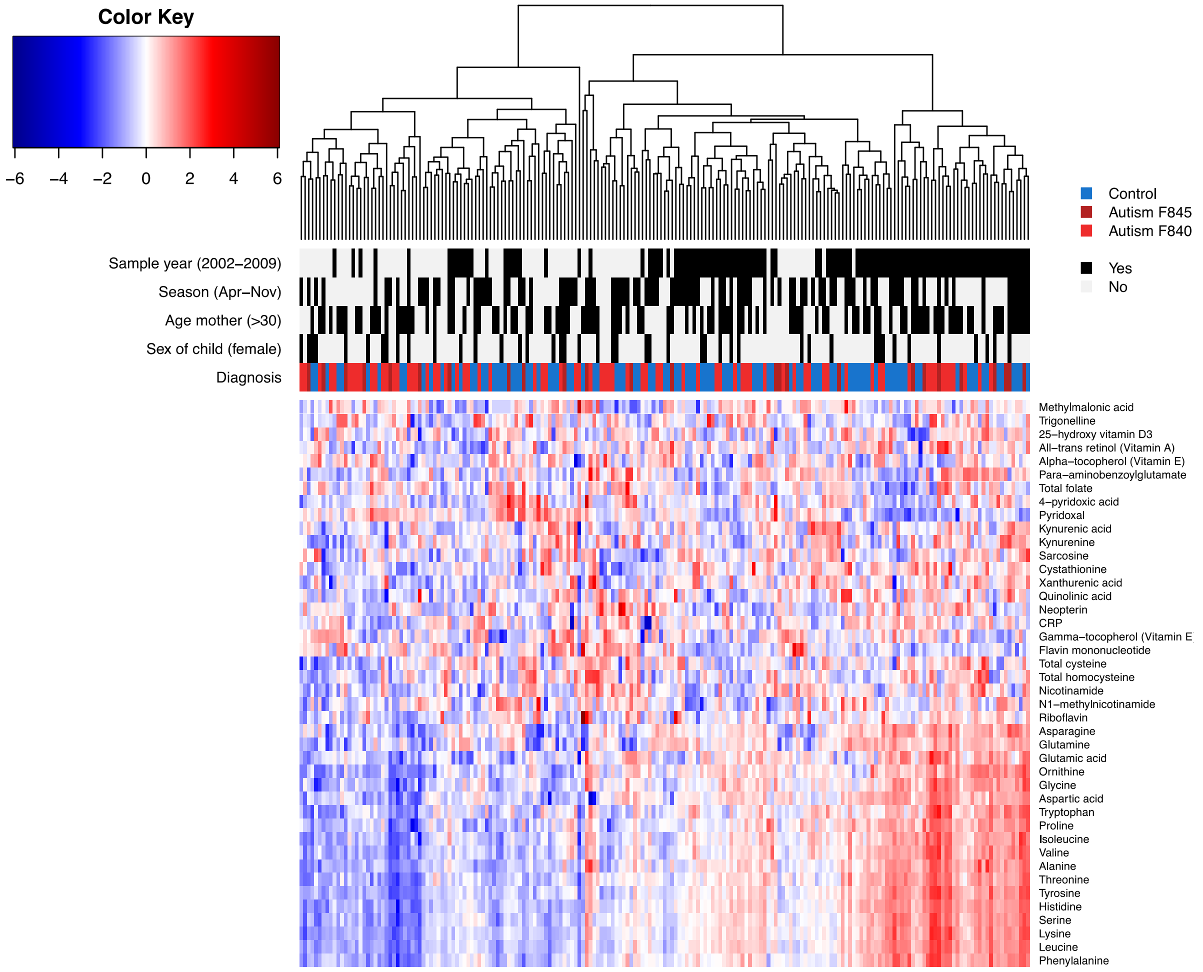

Supplementary Figure 3. Biomarker levels in each study participant, with a hierarchical cluster analysis based on Euclidian distances with complete linkage. Each column represents a participant, and each row a biomarker. Heat map colors represent standardized log-transformed biomarker levels, ranging from above the mean biomarker levels in all participants (red) and below mean biomarker levels (blue).
