# Supplementary material for: Maternal blood folate status during early pregnancy and occurrence of autism spectrum disorder in offspring: a study of 62 serum biomarkers
Source: Mol Autism. 2020 Jan 16;11:7. doi: 10.1186/s13229-020-0315-z (PMC6964211; doi:10.1186/s13229-020-0315-z)
Supplement: Supplementary file 4 — Additional file 4: Table S1. List of serum metabolites that were analyzed and used in further analysis. 1Only sum of THM and mTHM were included in the analysis as ‘total folate’. 2The sums of the Asparagine and Aspartic acid and the f Glutamine and Glutamic acid levels were used in statistical analysis because of conversion between this forms in the stored samples. In the whole 45 biomarkers could be identified and included in the analysis. [file 13229_2020_315_MOESM4_ESM.docx]

**Additional file 4: Table S1.** List of serum metabolites that were analyzed and used in further analysis.

| \| **Biomarker's name** \| number of samples, where the biomarker was detected \| \| notes \| further statistical analyses  yes(+)/no(-) \| \| --- \| --- \| --- \| --- \| --- \| \|  \| case grope \| control grope \|  \| \| **Folates and folate-catabolites:** \|  \|  \|  \|  \| \| 5-^1^methyl-tetrahydrofolate (mTHM)^1^ \| 100 \| 100 \|  \| + \| \| 4-alfa-hydroxy-5-methyl-tetrahydrofolate (hmTHM)^1^ \| 100 \| 100 \|  \| + \| \| 5-formyl-tetrahydrofolat \| 14 \| 5 \| not detected in majority of samples \| - \| \| folic acid \| 22 \| 12 \| not detected in majority of samples \| - \| \| para-aminobenzoylglutamate \| 98 \| 99 \|  \| + \| \| Acetamidobenzoylglutamate \| 75 \| 60 \| not detected in 33% of samples \| - \| \| **B-vitamines:** \|  \|  \|  \|  \| \| pyridoxal 5'-phosphate (B6 vitamin) \| not detected \| not detected \|  \| - \| \| pyridoxine (B6 vitamin) \| 0 \| 0 \| levels lower than reference ranges  in all samples \| - \| \| pyridoxal (B6 vitamin) \| 100 \| 100 \| same samples show levels higher than reference ranges \| + \| \| 4-pyridoxic acid (B6 vitamin) \| 100 \| 100 \|  \| + \| \| thiamine (B1 vitamin) \| 0 \| 0 \| not detected, probably due to sample storage at -20ºC \| - \| \| thiamine-monophosphate (B1 vitamin) \| 0 \| 0 \| not detected, probably due to sample storage at -20ºC \| - \| \| riboflavin (B2 vitamin) \| 100 \| 100 \|  \| + \| \| flavin mononucleotide (B2 vitamin) \| 100 \| 100 \|  \| + \| \| nicotinic acid (B3 vitamin) \| 66 \| 73 \| in other samples levels are lower than reference ranges \| + \| \| Nicotinamide (B3 vitamin) \| 100 \| 100 \|  \| + \| \| N1-methylnicotinamide (B3 vitamin) \| 100 \| 100 \|  \| + \| \| **Pterines and Tryptophan metabolites:** \|  \|  \|  \|  \| \| tryptophan \| 100 \| 100 \| concentration in proves considers to be a bit increased compare to reference ranges \| + \| \| kynurenine \| 100 \| 100 \| concentration in proves considers to be a bit low compare to reference ranges \| + \| \| 3-hydroxykynurenine \| 0 \| 0 \| not detected, probably due to sample storage at -20ºC \| - \| \| tryptophan/kynurenine ratio \| 100 \| 100 \|  \| + \| \| neopterin \| 100 \| 100 \|  \| + \| \| cystathionine \| 100 \| 100 \| concentration in proves considers to be a bit low compare to reference ranges \| + \| \| kynurenic acid \| 100 \| 100 \|  \| + \| \| xanthurenic acid \| 100 \| 100 \|  \| + \| \| quinolinic acid \| 100 \| 100 \|  \| + \| \| anthranilic acid \| 0 \| 0 \| not detected, not quantified due to poor sample storage \| - \| \| 3-hydroxyanthranilic acid \| 0 \| 0 \| not detected, probably due to sample storage at -20ºC \| - \| \| picolinic acid \| 0 \| 0 \| not detected \| - \| \| **Nicotin metabolites:** \|  \|  \|  \|  \| \| cotinine (nicotin metabolite) \| 100 \| 100 \| detected levels in majority of samples were around zero \| - \| \| trans-3'-hydroxycotinine (nicotin metabolite) \| 100 \| 100 \| detected levels in majority of samples were around zero \| - \| \| **Alkaloids:** \|  \|  \|  \|  \| \| trigonelline \| 100 \| 100 \|  \| + \| \| **Peptide:** \|  \|  \|  \|  \| \| C-reactive protein (CRP) \| 100 \| 100 \|  \| + \| \| **Other amino acids:** \|  \|  \|  \|  \| \| methylmalonic acid \| 100 \| 100 \| concentration was comparable to reference ranges \| + \| \| total homocysteine \| 100 \| 100 \| concentration was comparable to reference ranges \| + \| \| total cysteine \| 100 \| 100 \| concentration was comparable to reference ranges \| + \| \| methionine \| 54 \| 64 \| reduced to about 0-10% in the most samples, not detected in 41% of samples \| - \| \| serine \| 100 \| 100 \| concentration was much to high compare to reference ranges \| + \| \| glycine \| 100 \| 100 \| concentration was much to high compare to reference ranges \| + \| \| sarcosine \| 100 \| 100 \| concentration in proves considers to be a bit lower compare to reference ranges \| + \| \| histidine \| 100 \| 100 \| concentration in proves considers to be a bit increased compare to reference ranges \| + \| \| ornithine \| 100 \| 100 \| concentration in proves considers to be a bit increased compare to reference ranges \| + \| \| asparagine^2^ \| 100 \| 100 \| reduced to about 0-10% in the most samples compare to reference ranges \| + \| \| aspartic acid^2^ \| 100 \| 100 \| concentration was much to high compare to reference ranges \| + \| \| lysine \| 100 \| 100 \| concentration was much to high compare to reference ranges \| + \| \| alanine \| 100 \| 100 \| concentration was much to high  compare to reference ranges \| + \| \| isoleucine \| 100 \| 100 \| concentration in proves considers to be a bit increased compare to usual \| + \| \| leucine \| 100 \| 100 \| concentration was much to high compare to reference ranges \| + \| \| proline \| 100 \| 100 \| concentration in proves considers to be a bit increased compare to reference ranges \| + \| \| valine \| 100 \| 100 \| concentration in proves considers to be a bit increased compare to reference ranges \| + \| \| glutamine^2^ \| 100 \| 100 \| reduced to about 0-10% in the most samples compare to reference ranges \| + \| \| glutamic acid^2^ \| 100 \| 100 \| concentration was much to high compare to reference ranges \| + \| \| threonine \| 100 \| 100 \| concentration in proves considers to be a bit increased compare to reference ranges \| + \| \| tyrosine \| 100 \| 100 \| concentration in proves considers to be a bit increased compare to reference ranges \| + \| \| phenylalanine \| 100 \| 100 \| concentration was much to high compare to reference ranges \| + \| \| α-ketoglutaric acid \| 1 \| 0 \| could be detected in one sample \| - \| \| **Other vitamins:** \|  \|  \|  \|  \| \| alpha-tocopherol (Vitamin E) \| 100 \| 100 \|  \| + \| \| gamma-tocopherol (Vitamin E) \| 100 \| 100 \|  \| + \| \| all-trans retinol (Vitamin A) \| 100 \| 100 \|  \| + \| \| phylloquinone (vitamin K1) \| 100 \| 100 \| detected levels all samples were around zero \| - \| \| menaquinone-4 (vitamin K2) \| 100 \| 100 \| detected levels were around zero \| - \| \| 25-hydroxy vitamin D2 \| 100 \| 100 \| in one sample detected levels were above zero \| - \| \| 25-hydroxy vitamin D2 \| 100 \| 100 \|  \| + \| |
| --- | --- | --- | --- | --- | --- | --- | --- | --- | --- | --- | --- | --- | --- | --- | --- | --- | --- | --- | --- | --- | --- | --- | --- | --- | --- | --- | --- | --- | --- | --- | --- | --- | --- | --- | --- | --- | --- | --- | --- | --- | --- | --- | --- | --- | --- | --- | --- | --- | --- | --- | --- | --- | --- | --- | --- | --- | --- | --- | --- | --- | --- | --- | --- | --- | --- | --- | --- | --- | --- | --- | --- | --- | --- | --- | --- | --- | --- | --- | --- | --- | --- | --- | --- | --- | --- | --- | --- | --- | --- | --- | --- | --- | --- | --- | --- | --- | --- | --- | --- | --- | --- | --- | --- | --- | --- | --- | --- | --- | --- | --- | --- | --- | --- | --- | --- | --- | --- | --- | --- | --- | --- | --- | --- | --- | --- | --- | --- | --- | --- | --- | --- | --- | --- | --- | --- | --- | --- | --- | --- | --- | --- | --- | --- | --- | --- | --- | --- | --- | --- | --- | --- | --- | --- | --- | --- | --- | --- | --- | --- | --- | --- | --- | --- | --- | --- | --- | --- | --- | --- | --- | --- | --- | --- | --- | --- | --- | --- | --- | --- | --- | --- | --- | --- | --- | --- | --- | --- | --- | --- | --- | --- | --- | --- | --- | --- | --- | --- | --- | --- | --- | --- | --- | --- | --- | --- | --- | --- | --- | --- | --- | --- | --- | --- | --- | --- | --- | --- | --- | --- | --- | --- | --- | --- | --- | --- | --- | --- | --- | --- | --- | --- | --- | --- | --- | --- | --- | --- | --- | --- | --- | --- | --- | --- | --- | --- | --- | --- | --- | --- | --- | --- | --- | --- | --- | --- | --- | --- | --- | --- | --- | --- | --- | --- | --- | --- | --- | --- | --- | --- | --- | --- | --- | --- | --- | --- | --- | --- | --- | --- | --- | --- | --- | --- | --- | --- | --- | --- | --- | --- | --- | --- | --- | --- | --- | --- | --- | --- | --- | --- | --- | --- | --- | --- | --- | --- | --- | --- | --- | --- | --- | --- | --- | --- | --- | --- | --- | --- | --- | --- | --- | --- | --- | --- | --- | --- | --- | --- | --- | --- | --- | --- | --- | --- | --- | --- | --- | --- | --- | --- | --- | --- | --- | --- | --- | --- | --- | --- | --- | --- | --- | --- | --- | --- | --- | --- | --- | --- | --- | --- | --- | --- | --- | --- | --- |

^1^ Only sum of THM and mTHM were included in the analysis as ‘total folate’.

^2^ The sums of the Asparagine and Aspartic acid and the f Glutamine and Glutamic acid levels were used in statistical analysis because of conversion between this forms in the stored analytes.

In the whole 45 biomarkers could be identified and included in the analysis.
