# Supplementary material for: Maternal blood folate status during early pregnancy and occurrence of autism spectrum disorder in offspring: a study of 62 serum biomarkers
Source: Mol Autism. 2020 Jan 16;11:7. doi: 10.1186/s13229-020-0315-z (PMC6964211; doi:10.1186/s13229-020-0315-z)
Supplement: Supplementary file 5 — Additional file 5: Table S2. Differences of biomarkers levels between mothers of children with infantile autism and Asperger’s syndrome. [file 13229_2020_315_MOESM5_ESM.docx]

**Additional file 5: Table S2.** Differences of biomarkers levels between mothers of children with infantile autism and Asperger’s syndrome.

|  | **Infantile autism (F84.0)** | | | | | **Asperger's syndrome (F84.5)** | | | | | |
| --- | --- | --- | --- | --- | --- | --- | --- | --- | --- | --- | --- |
| **Biomarker** | **Median controls** | **Median** | **OR (95% CI)** | **P** | **P FDR adjusted** | **Median** | **OR (95% CI)** | **P** | **P FDR adjusted** | **P-heterogeneity^a^** | **P-heterogeneity FDR adjusted** |
| 4-pyridoxic acid | 18.2 | 18.5 | 0.69 (0.48-1.00) | 0.05 | 0.66 | 21.9 | 1.39 (0.92-2.10) | 0.12 | 0.73 | 0.01 | 0.44 |
| Kynurenine | 1.3 | 1.2 | 0.83 (0.60-1.15) | 0.25 | 0.59 | 1.3 | 1.64 (1.03-2.60) | 0.04 | 0.96 | 0.02 | 0.44 |
| Sarcosine | 0.84 | 0.74 | 0.78 (0.56-1.07) | 0.13 | 0.89 | 0.93 | 1.26 (0.79-1.99) | 0.33 | 0.96 | 0.09 | 0.96 |
| Quinolinic acid | 346 | 340 | 0.82 (0.59-1.13) | 0.22 | 0.88 | 371 | 1.28 (0.84-1.97) | 0.25 | 0.96 | 0.10 | 0.96 |
| 25-hydroxy vitamin D3 | 66.6 | 61.9 | 0.85 (0.62-1.18) | 0.34 | 0.59 | 60.0 | 0.57 (0.36-0.91) | 0.02 | 0.96 | 0.16 | 0.96 |
| Proline | 293 | 282 | 0.82 (0.60-1.14) | 0.24 | 0.89 | 316 | 1.19 (0.75-1.89) | 0.45 | 0.96 | 0.19 | 0.96 |
| Alanine | 633 | 594 | 0.97 (0.70-1.35) | 0.87 | 0.66 | 720 | 1.39 (0.89-2.18) | 0.15 | 0.99 | 0.20 | 0.96 |
| Ornithine | 142 | 131 | 0.88 (0.64-1.21) | 0.43 | 0.89 | 163 | 1.21 (0.77-1.91) | 0.41 | 0.99 | 0.26 | 0.96 |
| Glutamine | 83.0 | 61.4 | 0.95 (0.69-1.32) | 0.76 | 0.66 | 55.4 | 0.71 (0.47-1.09) | 0.12 | 0.99 | 0.29 | 0.96 |
| Pyridoxal | 15.1 | 15.6 | 0.90 (0.64-1.27) | 0.55 | 0.89 | 16.8 | 1.21 (0.75-1.95) | 0.43 | 0.99 | 0.31 | 0.96 |
| Cystathionine | 0.08 | 0.07 | 0.94 (0.68-1.29) | 0.70 | 0.66 | 0.07 | 0.71 (0.44-1.13) | 0.14 | 0.99 | 0.32 | 0.96 |
| Riboflavin | 41.6 | 41.8 | 0.77 (0.55-1.09) | 0.14 | 0.98 | 44.1 | 1.01 (0.67-1.51) | 0.98 | 0.96 | 0.32 | 0.96 |
| Trigonelline | 0.31 | 0.26 | 0.85 (0.59-1.22) | 0.37 | 0.66 | 0.28 | 0.63 (0.37-1.07) | 0.09 | 0.96 | 0.37 | 0.96 |
| Kynurenic acid | 35.7 | 32.7 | 0.70 (0.50-0.98) | 0.04 | 0.89 | 35.0 | 0.89 (0.56-1.40) | 0.61 | 0.73 | 0.40 | 0.96 |
| Methylmalonic acid | 0.16 | 0.15 | 1.03 (0.74-1.42) | 0.87 | 0.88 | 0.17 | 1.29 (0.83-2.00) | 0.26 | 0.99 | 0.41 | 0.96 |
| Alpha-tocopherol (Vitamin E) | 18.9 | 17.9 | 0.86 (0.62-1.18) | 0.34 | 0.93 | 18.1 | 1.07 (0.68-1.68) | 0.78 | 0.96 | 0.43 | 0.96 |
| Glutamic acid | 461 | 465 | 1.19 (0.86-1.66) | 0.30 | 0.66 | 509 | 1.49 (0.94-2.37) | 0.09 | 0.96 | 0.43 | 0.96 |
| Tryptophan | 89.3 | 87.9 | 1.01 (0.73-1.40) | 0.96 | 0.89 | 87.7 | 1.26 (0.80-1.96) | 0.32 | 0.99 | 0.43 | 0.96 |
| All-trans retinol (Vitamin A) | 1.6 | 1.5 | 0.92 (0.66-1.28) | 0.60 | 0.89 | 1.5 | 1.15 (0.72-1.83) | 0.57 | 0.99 | 0.44 | 0.96 |
| Isoleucine | 103 | 96 | 0.97 (0.70-1.35) | 0.88 | 0.89 | 106 | 1.18 (0.75-1.87) | 0.47 | 0.99 | 0.49 | 0.96 |
| Total homocysteine | 6.9 | 7.0 | 1.18 (0.85-1.63) | 0.33 | 0.95 | 6.5 | 0.97 (0.61-1.56) | 0.91 | 0.96 | 0.51 | 0.96 |
| Methionine | 1.2 | 1.2 | 0.92 (0.59-1.43) | 0.71 | 0.89 | 1.5 | 1.18 (0.62-2.27) | 0.61 | 0.99 | 0.52 | 0.96 |
| Valine | 347 | 324 | 0.90 (0.64-1.24) | 0.51 | 0.93 | 339 | 1.06 (0.68-1.67) | 0.79 | 0.99 | 0.54 | 0.96 |
| CRP | 2.5 | 2.9 | 1.17 (0.85-1.61) | 0.35 | 0.76 | 3.4 | 1.39 (0.85-2.27) | 0.19 | 0.96 | 0.56 | 0.96 |
| Xanthurenic acid | 35.2 | 31.9 | 0.81 (0.58-1.12) | 0.20 | 0.66 | 29.6 | 0.68 (0.43-1.09) | 0.11 | 0.96 | 0.56 | 0.96 |
| Leucine | 313 | 272 | 0.96 (0.69-1.33) | 0.80 | 0.89 | 274 | 1.13 (0.71-1.78) | 0.61 | 0.99 | 0.57 | 0.96 |
| Lysine | 314 | 288 | 1.00 (0.72-1.40) | 0.99 | 0.89 | 293 | 1.17 (0.74-1.84) | 0.50 | 0.99 | 0.59 | 0.96 |
| Serine | 347 | 338 | 1.03 (0.74-1.43) | 0.86 | 0.89 | 334 | 1.19 (0.75-1.88) | 0.47 | 0.99 | 0.62 | 0.97 |
| Phenylalanine | 172 | 169 | 1.07 (0.77-1.49) | 0.69 | 0.89 | 179 | 1.21 (0.77-1.91) | 0.41 | 0.99 | 0.66 | 0.99 |
| Tyrosine | 90.2 | 81.5 | 0.94 (0.67-1.31) | 0.70 | 0.94 | 84.5 | 1.04 (0.66-1.65) | 0.86 | 0.99 | 0.71 | 0.99 |
| Glycine | 463 | 440 | 1.04 (0.75-1.44) | 0.82 | 0.89 | 462 | 1.15 (0.73-1.82) | 0.55 | 0.99 | 0.72 | 0.99 |
| Neopterin | 7.0 | 7.0 | 0.99 (0.73-1.36) | 0.97 | 0.92 | 7.2 | 0.91 (0.58-1.43) | 0.67 | 0.99 | 0.74 | 0.99 |
| Gamma-tocopherol (Vitamin E) | 1.7 | 1.7 | 1.00 (0.73-1.38) | 0.99 | 0.93 | 1.7 | 1.09 (0.69-1.73) | 0.71 | 0.99 | 0.76 | 0.99 |
| Histidine | 155 | 141 | 0.98 (0.70-1.36) | 0.89 | 0.93 | 130 | 1.06 (0.67-1.69) | 0.79 | 0.99 | 0.77 | 0.99 |
| Aspartic acid | 201 | 196 | 0.98 (0.71-1.35) | 0.91 | 0.93 | 181 | 0.92 (0.59-1.44) | 0.73 | 0.99 | 0.83 | 0.99 |
| Total cysteine | 209 | 213 | 1.19 (0.86-1.64) | 0.29 | 0.89 | 213 | 1.25 (0.80-1.96) | 0.33 | 0.96 | 0.86 | 0.99 |
| Nicotinamide | 272 | 267 | 1.00 (0.73-1.36) | 0.98 | 0.94 | 246 | 1.03 (0.66-1.61) | 0.88 | 0.99 | 0.89 | 0.99 |
| Asparagine | 15.8 | 13.1 | 1.02 (0.74-1.41) | 0.89 | 0.93 | 14.4 | 1.06 (0.67-1.67) | 0.80 | 0.99 | 0.90 | 0.99 |
| Flavin mononucleotide | 8.1 | 7.7 | 0.81 (0.58-1.12) | 0.20 | 0.89 | 7.9 | 0.83 (0.52-1.31) | 0.42 | 0.96 | 0.92 | 0.99 |
| Para-aminobenzoylglutamate | 0.86 | 0.86 | 0.93 (0.64-1.34) | 0.69 | 0.94 | 1.07 | 0.95 (0.56-1.61) | 0.86 | 0.99 | 0.93 | 0.99 |
| Acetamidobenzoylglutamate | 0.53 | 0.57 | 1.15 (0.76-1.73) | 0.50 | 0.89 | 0.68 | 1.17 (0.68-2.02) | 0.57 | 0.99 | 0.96 | 0.99 |
| Threonine | 227 | 220 | 0.97 (0.70-1.34) | 0.84 | 0.95 | 233 | 0.98 (0.62-1.55) | 0.93 | 0.99 | 0.96 | 0.99 |
| N1-methylnicotinamide | 121 | 122 | 0.90 (0.66-1.24) | 0.53 | 0.89 | 100 | 0.90 (0.57-1.40) | 0.63 | 0.99 | 0.97 | 0.99 |
| Total folate* | 10.7 | 12.8 | 1.69 (1.20-2.37) | 0.003 | 0.59 | 14.0 | 1.68 (1.05-2.69) | 0.03 | 0.13 | 0.99 | 0.99 |
| OR: Odds ratios, CI: confidence interval | | | | | | | | | | | |
| ^a^ Wald's test of heterogeneity in type-specific ORs from multinomial logistic regression models | | | | | | | | | | | |
| ^*^  the sum of 5-methyl-tetrahydropholate and 4-alfa-hydroxy-5-methyl-tetrahydrofolate | | | | | | | | | | | |
